# Leptospiral Infection, Pathogenesis and Its Diagnosis—A Review

**DOI:** 10.3390/pathogens10020145

**Published:** 2021-02-01

**Authors:** Antony V. Samrot, Tan Chuan Sean, Karanam Sai Bhavya, Chamarthy Sai Sahithya, SaiPriya Chan-drasekaran, Raji Palanisamy, Emilin Renitta Robinson, Suresh Kumar Subbiah, Pooi Ling Mok

**Affiliations:** 1 School of Bioscience, Faculty of Medicine, Bioscience and Nursing, MAHSA University, Jenjarom, Selangor 42610, Malaysia; tanchuansean@gmail.com; 2 Department of Biotechnology, School of Bio and Chemical Engineering, Sathyabama Institute of Science and Technology, Jeppiaar Nagar, Chennai, Tamil Nadu 627 011, India; saibhavyakaranam@gmail.com (K.S.B.); saisahithya1998@gmail.com (C.S.S.); saipriya24c@gmail.com (S.C.); raji.naomi10@gmail.com (R.P.); 3 Department of Food Processing Technology, Karunya Institute of Technology and Science, Coimbatore, Tamil Nadu 641 114, India; emilinrenitta@gmail.com; 4 Department of Medical Microbiology and Parasitology, Faculty of Medicine and Health Sciences, Universiti Putra Malaysia, UPM Serdang, Selangor 43400, Malaysia; sureshkudsc@gmail.com; 5 Department of Biotechnology, Bharath Institute of Higher Education and Research (BIHER), Selaiyur, Tamil Nadu 600 073, India; 6 Genetics and Regenerative Medicine Research Centre, Faculty of Medicine and Health Sciences, Universiti Putra Malaysia, UPM Serdang, Selangor 43400, Malaysia; 7 Department of Biomedical Science, Faculty of Medicine and Health Sciences, Universiti Putra Malaysia, UPM Serdang, Selangor 43400, Malaysia; 8 Department of Clinical Laboratory Sciences, College of Applied Medical Sciences, Jouf University, Sakaka P.O. Box 2014, Aljouf Province, Saudi Arabia

**Keywords:** leptospirosis, leptospiral proteins, pathogenesis, diagnosis

## Abstract

Leptospirosis is a perplexing conundrum for many. In the existing literature, the pathophysiological mechanisms pertaining to leptospirosis is still not understood in full. Considered as a neglected tropical zoonotic disease, leptospirosis is culminating as a serious problem worldwide, seemingly existing as co-infections with various other unrelated diseases, including dengue and malaria. Misdiagnosis is also common as non-specific symptoms are documented extensively in the literature. This can easily lead to death, as the severe form of leptospirosis (Weil’s disease) manifests as a complex of systemic complications, especially renal failure. The virulence of *Leptospira* sp. is usually attributed to the outer membrane proteins, including LipL32. With an armament of virulence factors at their disposal, their ability to easily adhere, invade and replicate within cells calls for a swift refinement in research progress to establish their exact pathophysiological framework. As an effort to reconstitute the current knowledge on leptospirosis, the basis of leptospiral infection, including its risk factors, classification, morphology, transmission, pathogenesis, co-infections and clinical manifestations are highlighted in this review. The various diagnostic techniques are also outlined with emphasis on their respective pros and cons.

## 1. Introduction

Infectious diseases are caused by a wide variety of pathogens, including bacteria, fungi, parasites and viruses. These microorganisms have the ability to transfer from one host to another, potentially culminating into worldwide pandemics. The infective capability of such microorganisms is augmented by a multitude of factors, especially the migratory behaviour of populations across various countries [1]. Recent improvements in preventive and therapeutic regimens have inadvertently established the false notion that infectious diseases are not significant threats to public health, but in reality, they still persist as one of the major causes of high morbidity and mortality rates every year [2]. Infectious diseases by means of animal or vector transmission are known as zoonoses. Strictly speaking, zoonoses involves a particular pathogen that transmits from an animal (non-human) to a human host [3]. A global-wide analysis from 1940 to the early 20th century found that 60.3% of emerging infectious diseases (EIDs) were caused by fast-growing zoonotic pathogens [4,5]. One of the most notable zoonotic infections is leptospirosis, which is caused by *Leptospira* sp. [6]. Based on its thin structure and spiral shape, the term “*Leptospira*” was first coined by Noguchi [6] who suggested the term to be put forward as a new genus. In Brazil, a study by Mayer et al. [7] evidently demonstrated the presence of *Leptospira* sp. in bats (one of the many vectors of leptospirosis), further reinforcing the association of *Leptospira* to its epidemiological data in the existing literature.

According to the Leptospirosis Burden Epidemiology Reference Group (LERG), the risk factors for leptospirosis increases due to rainfall, flooding, open sewers, crowding, populace, animal contacts and poor sanitation [8]. Recent studies have demonstrated that the outbreak of leptospirosis in Malaysia and Brazil occurred after major floods [9,10]. This reinforces the tendency for leptospirosis to culminate into a worldwide outbreak as several countries (in addition to Malaysia and Brazil) are prone to the after effects of global warming and severe floods. Besides that, the prevalence of outbreaks is highly associated with various outdoor activities, such as wildlife recreational programs, adventure travels and army expeditions [11]. To reinforce, a study by Dierks et al. [12] reported that US marine trainees situated in Japan were found to be inflicted with leptospirosis, as they were constantly exposed to stagnant waters in a simulated jungle warfare environment. Since warm and humid condition incites the transmission of leptospirosis [13], outbreaks typically occur in tropical areas [14] and sometimes during summer or fall in temperate regions. It is also estimated that approximately 500,000 high-risk cases occur globally with a 30% fatality rate per annum [15]. Recent studies have demonstrated the global incidence of leptospirosis, as evidently demonstrated in Italy [16], Pakistan [17], Japan [18], Brazil [19], India [20] and Sri Lanka [21]. Records of epidemics were found widely spread between 2000 to 2010 in Nicaragua, Sri Lanka and Philippines [14].

With a case fatality ratio of 26.89 out of 7587 cases in 10 years [2], leptospirosis still remains as one of the major health concerns worldwide. However, the false notion regarding the severity and global impact of leptospirosis hinders the worldwide surveillance, control and detection of the disease. Hartskeerl et al. [22] emphasized that the mortality and morbidity rates of leptospirosis are significantly underestimated due to the lack of notification and epidemiological effort in various countries. Moreover, only those with severe leptospirosis are taken into account for the estimation of the disease’s incidence rate. The lack of knowledge on leptospirosis worldwide worsens the aforementioned problem. As an effort to reconstitute the current knowledge on leptospirosis, the present review discusses the disease from all angles pertaining to its risk factors, causative agent, pathogenesis, clinical manifestations and diagnostic techniques.

## 2. Risk Factors

Occupation, migratory behaviour, gender and age are all significant risk factors of leptospirosis. In the past, leptospirosis was first considered as an occupational disease, whereby coal miners were the first occupational risk groups to be documented in the literature [23]. In all actuality, various mammals including feral, farm and pet animals harbours the disease-causing agent. This extends the occupational risk to wider lengths as it is capable of infecting farmers, miners, slaughterhouse laborers, pet traders, veterinarians, rodent catchers, sewer workers, garbage collectors and livestock ranchers, as these job scopes have continuous and constant contact with various animals throughout their duty [14,22]. Although they are not considered as occupational risk factors, water-based sports [24] and international travels [22] are known to contribute significantly to the rapid transmission of leptospirosis.

Due to the rising risk of leptospirosis in various parts of the world, the World Health Organization (WHO) initiated the Leptospirosis Burden Epidemiology Reference Group (LERG) which aims to assess the overall population health by quantifying the morbidity and mortality rates due to leptospirosis [25]. A report submitted from the second LERG meeting stated that the median case-fatality percentage was significantly higher in women as compared to men [26]. However, that does not mean that women are more likely to be infected with the disease. From the same report, men are actually more likely to be infected with leptospirosis as they are more prone to occupational exposure in outdoor settings. For men, the median incidence of the disease was the highest for those older than the age of 59, followed by those between the ages of 20 to 29. For women, approximately 37% of leptospirosis cases were reported for those aged between 40 and 49 [26]. As evident from the aforementioned data, it can be inferred that both gender and age play a significant role in the occurrence and fatality of leptospirosis.

## 3. Classification of *Leptospira*

**Figure 1:**
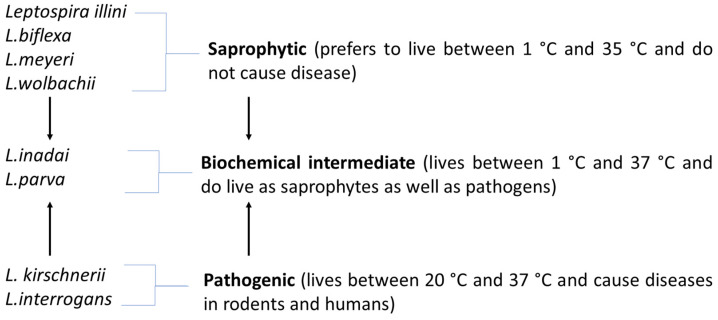
Illustration depicting the relationship between pathogenic, intermediate and saprophytic *Leptospira* and the distinct differences between them.

Leptospires are classified into various serovars based on the distinct expression of surface-exposed epitopes in a mosaic of their lipopolysaccharide (LPS) antigens [27]. Initially, *Leptospira* was only classified into *L. interrogans* and *L. biflexa,* which clearly divides the pathogenic and non-pathogenic species. Later on, these two classifications were further divided into specific serovars based on the presence of homologous antigens (nearly 60 serovars under *L. biflexa* and at least 225 serovars under *L. interrogans*). As the years go by, at least 21 more species have been identified under *Leptospira* with more than 200 specific serovars [27,28]. In all actuality, the classification of *Leptospira* is vast and more sophisticated than it seems. The present review concentrates on the three basic classifications of *Leptospira* (Figure 1) according to their capability to cause disease.

Even though the saprophytic and pathogenic types of *Leptospira* have some similarities in their structure and genetic makeup, they vary by other factors such as the ability to cause disease and temperature withstanding capacity. Saprophytic leptospires are capable of growing at low temperatures (5–35 °C), found naturally in soil and water but they do not have the capability to cause any infections. The first saprophytic leptospire to be sequenced (also the major saprophytic species) is *Leptospira biflexa*. It consists of a large number of serovars and they are usually found on moist soils and on water surfaces (rarely found in humans and other animals) [28,29]. Other saprophytic leptospires include *L. meyeri, L. wolbachii, L. yanagawae, L. vanthielii* and *L. terpstrae* [28]. Similarly like *L.biflexa*, the aforementioned organisms are not capable of inflicting diseases. Although it is rare, saprophytic leptospires were documented to be found in the urine of various mammals. These organisms are distinctively identified by the presence of two bundles of cytoplasmic tubules as well as flagella, with their basal structures resembling that of Gram-positive bacteria [30]. As these organisms have somewhat similar structures with other species under the same genus, the ability to easily manipulate and control saprophytic leptospires makes them excellent and safer alternatives (as compared to pathogenic leptospires) for structural studies [31].

Intermediate leptospires exist as the biochemical intermediate of saprophytic and pathogenic leptospires One major leptospire from this group is *Leptospira parva,* which is considered as the biochemical intermediate of *L. interrogans* and *L. biflexa* [29]. *L. parvais* capable of co-existing with various saprophytic and pathogenic leptospires [32]. With more than 5 identified species from this group [28], other notable intermediate leptospires include *L. broomi, L. inadai, L. licerasiae, L. wolffii and L. fainei* [33]. Pathogenic leptospires require temperatures ranging from 20–35 °C, are usually found in rodents and have flagella with basal structures resembling that of Gram-negative bacteria [29]. Compared to saprophytic leptospires, the leptospires from this group are more significant to healthcare. This is because the various members of this group, including *L. interrogans, L. weilii*, *L. noguchii*, *L. borgpetersenii* [34], *L. kirschneri* and *L. santarosai* [19] are capable of inflicting leptospirosis, subsequently influencing morbidity and mortality rates.

## 4. Morphology

Leptospires are aerobic and slow-growing organisms that are highly susceptible towards drought and hypertonic conditions [35]. Attributed to various ocular manifestations [36,37], these organisms are helical-shaped with distinct hook-ends that allows them to be clearly differentiated from other spirochaetes. In terms of their size, they are thin and long with a thickness of about 0.1 to 0.15 μm and 6 to 20 μm in length [24]. Jutras et al. [38] documented the ability of leptospires to elongate in a lateral fashion by synthesizing peptidoglycan for growth. Collectively, the aforementioned morphological features enable leptospires to easily burrow into tissues of their victims. Jackson et al. [31] demonstrated that the presence of bactofilins in *L. biflexa* are responsible for the retainment of the organism’s helical shape. Bactofilins were also hypothesized to have played a role in the rapid movement of the organism, but as of date their exact functions are yet to be determined. The chromosomal DNA of leptospires is distributed along the length of the cell, rather than the center [39]. Like most Gram-negative bacteria, *Leptospira* spp. consists of an outer membrane with various functional proteins. Uniquely, they also consist of periplasmic flagella that allows the bacteria to be motile. As these morphological features contribute significantly to the clinical importance of *Leptospira*, they are selectively discussed in further in detail as following.

### 4.1. Cell Outer Membrane Structure and Its Proteins

*Leptospira* consists of an outer membrane (majorly composed of LPS) that is quite similar to that of *Brachyspira*, allowing them to stand out from other spirochaetes such as *Treponema* and *Borrelia* [28,40]. Although the outer membranal structure of leptospires adopts similar characteristics from both Gram-negative and Gram-positive bacteria, the endotoxic potential of Leptospiral LPS is significantly lower as compared to the average Gram-negative LPS [41]. This allows us to infer that other proteinaceous structures found on the outer membrane of leptospires contributes more to their virulence and pathogenicity. When leptospires are suspended in distilled water and subsequently dried, they are capable of forming a sheath-like structure around their periphery [42]. This was earlier considered to be the after effects of bacterial washing and the mere observation of cell wall-like structures of *Leptospira*. This hypothesis was later reinforced by Anderson et al. [43], as they found the outer sheath and the wall membrane are important control mechanisms for the viability and permeability of leptospires. From the aforementioned findings, it is evident that the outer membrane of leptospires is functionally versatile due to the complex composition of the proteinaceous components found there. As an effort to better understand the multifold functions of *Leptospira*’s outer membrane, the following subsections review and distinguish the roles of various leptospiral outer membrane proteins described in the existing literature.

#### 4.1.1. Outer Membrane Proteins

**Figure 2:**
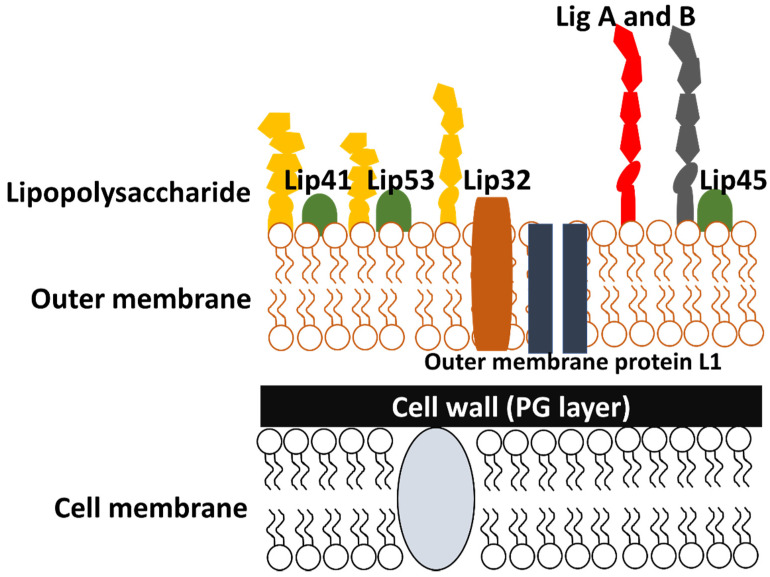
Illustration depicting the outer layers of *Leptospira* species, highlighting the various proteins found on the outer membrane.

**Figure 3:**
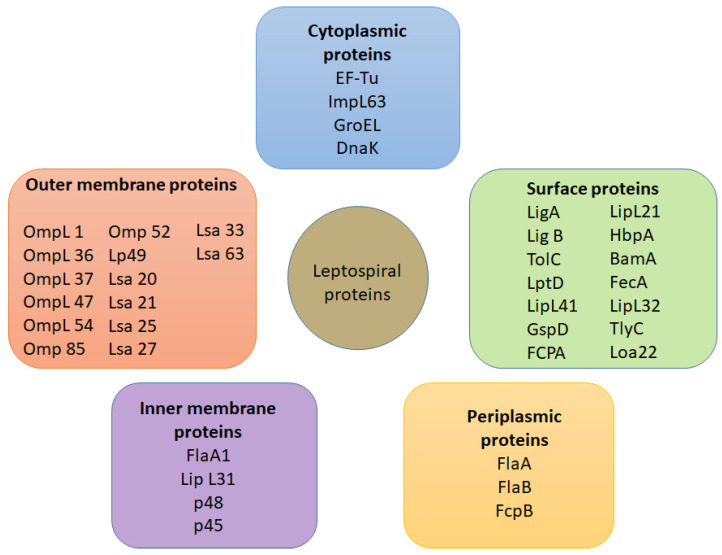
Pictorial representation of notable leptospiral proteins [44,51,52,57,58,59,60,61,62,63,64,65,66,67,68,69,70,71,72,73,74,75,76,77,78].

Unlike other spirochaetes, leptospires have a distinct outer membrane that consists of LPS with various beta barrel transmembrane proteins [44] (Figure 2). Typically, the outer membrane proteins functionally act as diffusion barriers, as well as being involved in the process of septum formation and nutrient uptake for growth [45]. Despite these classical life-sustaining functions, these proteins were also shown to demonstrate some level of virulence against various host mechanisms as they are usually engaged in scuffles with host defence units (since they are directly exposed to host environment in the exterior) [46]. Lipoprotein L32 (LipL32) [47], lipopolysaccharides (LPS) [48], *Leptospira* immunoglobulin-like proteins (Lig) [49], *Leptospira* endostatin-like proteins (Len) [50,51] and *Leptospira* OmpA-like lipoprotein (Loa22) [52] are well-established in the existing literature due to their highly virulent nature. With the help of various surfactants, a multitude of approaches were successfully carried out to singly isolate the aforementioned proteins, allowing them to be successfully classified into five major protein classes (Figure 3) [53,54,55,56,57,58,59,60,61,62,63,64,65,66,67,68,69,70,71,72,73,74,75,76,77,78].

#### 4.1.2. LipL32

Lipoprotein L32 (LipL32) is the most abundant outer membrane protein found in *Leptospira* species. It is a lipoprotein with a single tag at its N-terminal [57,79,80] and specific lipid-based modification at its Cys residue [81]. Various affinity-based studies have found that the intermediate domain and C-terminal of LipL32 are vital for its interaction with a multitude of components in the extracellular matrix (ECM), including laminin, collagen and fibronectin [82,83]. In terms of its virulence, Witchell et al. [84] confirmed that post translational modification of LipL32 is related to host innate immunity as it directly induces host inflammatory response. This most often leads to tubulointerstitial nephritis as large amounts of inflammatory mediators, including monocyte chemoattractant protein-1 (MCP-1), RANTES, inducible nitric oxide synthase (iNOS) and tumour necrosis factor-α (TNF-α) are produced in the vicinity of renal cells [47]. In addition, LipL32 also acts as a haemolysin, which in turn induces proinflammatory cytokines through the various toll-like receptor (TLR) signalling pathways [85]. As established in the existing literature, there are generally two types of TLRs (TLR2 and TLR4) involved in leptospirosis, but Yang et al. [86] accentuated the pathogenic significance of TLR2 as it directly interacts with LipL32. Lo et al. [87] also found that the calcium-binding cluster (consisting of several essential residues such as Asp, Thr and Tyr) present on LipL32 is responsible for sustaining LipL32 conformation for proper TLR2-mediated inflammatory responses in host renal cells. In terms of their diagnostic applications, LipL32 is used as a target gene in multiplex polymerase chain reaction (PCR) which significantly improves the diagnostic sensitivity and specificity of leptospiral infections [88]. Sumarningsih et al. [89] reported the use of recombinant LipL32 as an antigen in enzyme-linked immunosorbent assay (ELISA), allowing for a safer and easier approach in leptospiral diagnosis.

#### 4.1.3. OmpL1

Outer membrane protein L1 (OmpL1) is a transmembrane protein with 320 amino acids that functionally acts as a porin (diffusion channels) in all pathogenic species of *Leptospira* [90,91]. The *ompL1* gene, which encodes for the OmpL1 protein, is divided into three groups (*ompL1/1*, *ompL1/2* and *ompL1/3*) based on the molecular phylogenetic relationship of their amino acid and nucleotide sequences [91]. As reported by Dong et al. [91], all three of these genes were found in pathogenic leptospires in China, whereby the distinct protein products are naturally expressed on the surface of the leptospires. OmpL1 acts as an ECM-binding protein that commonly binds to fibronectin and laminin, and is also capable of interacting with plasminogen (PLG) as its receptor [60,92]. Through in vivo studies, Fernandes et al. [60] described the capability of OmpL1 in promoting the proliferation of lymphocytes and stimulating the release of cytokines [93] from T-helper 1 and T-helper 2 cells. These findings were reinforced by a study done by Haake et al. [94], in which they found that OmpL1 provided a significant level of immune protection effect, paving the way for vaccine production against *Leptospira*. In diagnosis, the high percentage of true-positive leptospirosis cases (microscopic agglutination test-positive) is largely due to the high specificity of the IgG antibody-response towards OmpL1, enabling the clear differentiation from other diseases [60].

#### 4.1.4. Loa22

Loa22 is a 22 kDa bipartite leptospiral lipoprotein with a C-terminal OmpA domain (consisting of approximately 195 amino acids) and an N-terminal domain [52,95]. This lipoprotein has an atypical Leu residue prior to Cys with a single cleavage site between the 20th and 21st residues. By studying the amino acid sequence of the C-terminal OmpA domain, it is well-established that other leptospiral outer membrane proteins demonstrated sequence homology with OmpA. In addition, Koizumi et al. [95] found that Loa22 is a surface-exposed protein, corroborating with the more recent immunofluorescent-based findings by Ristow et al. [52].

#### 4.1.5. LigB

*Leptospira* immunoglobulin-like protein B (LigB) is capable of binding to several ECM components such as fibronectin, collagen, laminin fibrinogen and elastin. There are mainly three classes of leptospiral immunoglobin proteins—namely, LigA, LigB and LigC which possesses 13, 12 and 13 immunoglobulin-like domains, respectively [51,96,97]. The genes coding for the respective Lig proteins, *ligA, ligB* and *ligC* encodes the virulence determinants in pathogenic leptospires. As the most clinically significant of the three, *ligB* is present in almost all *Leptospira* species, including *L. weilii*, *L. interrogans, L. kirschneri, L. borgpetersenii* and *L. noguchii*, while *ligA* is present in only some species of *Leptospira*, such as *L. interrogans* and *L. kirschneri*. *ligC* is a pseudogene found in certain Leptospiral species, including *L. weilii, L. interrogans* and *L. kirschneri* [98]. In terms of their sequence homology, the amino acid sequences found on the N-terminals of LigA and LigB are exactly identical whereas the amino acid sequences found on their C-terminals are only 34% similar. Overall, LigC only shows 38% sequence homology with LigA and LigB, highlighting it as a fairly distant counterpart of the two Lig immunoglobulins [99,100]. The expression of these proteins is controlled by physiological osmolarity, which is well-acknowledged as a key environmental signal for leptospires to enhance their binding to host cells [101,102]. A study by Lin et al. [103] found that LigB consists of two variable regions (LigBCen and LigBCtv) that enables leptospires to bind effectively to host cells. In terms of their diagnostic value, various studies have documented the possibility of utilizing the Lig proteins as diagnostic markers for early diagnosis of leptospirosis [104] and also as a potential candidate for vaccine development [105].

#### 4.1.6. LenA

Leptospiral endostatin-like protein A (LenA) is also known as leptospiral surface adhesin protein (Lsa24) [106] and leptospiral factor H binding protein (LfhA) [107]. Lsa24 and LfhA are found to be the same protein and thus they are collectively renamed as a single entity: LenA. Verma et al. [107] reported that LenA is capable of interacting with human factor H-related protein 1 (FHR-1), which has a high degree of similarity with factor H [108]. Factor H is a fluid-phase regulator that plays a vital role during activation of the alternative complement pathway in host immune systems. As their outer membrane consists of LenA, pathogenic leptospires are capable of binding to factor H, consequently becoming resistant to complement mediated-killing via the alternative pathway. The gene sequence of LenA is highly conserved and are thought to encode for membrane associated lipoproteins in *Leptospira* [46]. Vieira et al. [109] reported that the lysine residues found on LenA has the ability to bind with plasminogen in a dose dependent manner. This in turn generates the enzymatically active plasmin on the leptospires’ surface, which can be detrimental to host tissues as plasmin can easily degrade ECM components (aligning with leptospiral host tissue penetration) [110].

### 4.2. Periplasmic Flagella

Contrary to most bacteria, *Leptospira* consists of two periplasmic flagellum (also known as axial filament and endoflagella) that are anchored to opposite ends of the organism and extends horizontally in the periplasmic space. Although periplasmic flagella primarily provide leptospires with the ability to move about via translational and non-translational movements [41], they also serve as cytoskeleton and maintains the flat-wavy shape of the organism [111]. Each of the periplasmic flagellum extends to a very short distance from their respective poles, visualized as distinct hook-like end [112]. Both extensions from opposite ends terminate at the central region of leptospires without touching each other [111]. Takabe et al. [111] found that the swimming manner of leptospires differ according to the viscosity of the media they are suspended in. In non-viscous media, the organism mainly utilizes their hook-like ends for motility support, while a screw-like motion is utilized in the case of viscous media. In terms of their morphological framework, leptospiral flagellum consists of three major parts: the filament, flexible hook and the basal complex [39].

In *L. pomona*, a typical filament is about 100–120 A in diameter [113]. In leptospires, this structure is connected to the basal complex with the help of a flexible hook. The hook-filament junction is composed of FlgK and FlgL proteins, which keeps the filaments affixed to their respective poles [39]. Both of the filaments extend from opposite ends of the organism and extend along the protoplasmic cylinder until it reaches the central region. Leptospires are thus capable of achieving motility via rotational movement of these filaments [24]. Each flagellar filament consists of a cylindrical core that is made up of four major subunits (FlaB1, FlaB2, FlaB3 and FlaB4) and are enclosed by an outer sheath made up of FcpA, FlaA1 and FlaA2 subunits [32,114]. The FlaB1 and FcpA subunits interact with one another to ensure that the cylindrical core and the outer sheath carry out their contractional actions in a concerted fashion. Various literatures have found that the thickness of the outer sheath largely depends upon the identity of the leptospires (Table 1).

Raddi et al. [117] discovered that there are novel periplasmic filaments (PFils) existing in the periplasmic space. With the help of cryo-electron tomography (cryo-ET), PFils were found to be smaller than flagellar filaments (approximately 8 vs. 22 nm). Evidently, the PFils were found to be wrapping around the organism’s cell body, especially in the central region that is devoid of periplasmic flagella. Additionally, Sasaki et al. [72] discovered that FcpA interacts with FlaA2 to produce the coiling force of PFils, further potentiating the organism’s movement capabilities.

The basal complex acts as a transmembrane rotary motor, contributing significantly to the motility of *Leptospira* as it allows the flagellar filaments (bridged by the hook) to rotate while remaining affixed to the same axis [39]. In terms of their morphological framework, it is well-established that the basal complex of leptospiral flagella is fairly similar to that of Gram-negative bacteria [32]. It is made up of various subunits, including the rod (directly attached to the hook), L ring, P ring and MS (membrane/supramembrane) ring. In actuality, the basal complex in leptospires is much larger and sophisticated compared to other common bacterial models (such as *Escherichia coli*). Raddi et al. [117] documented that leptospiral basal complex consists of additional subunits, including a flagellar C ring, an export apparatus as well as a stator. The basal complex of leptospires also consists of a collar, briefly described as a large and complex structure that is anchored to the MS ring and the inner membrane [118]. Moon et al. [119] suggested that the collar (specifically collar protein FlbB) may be vital for leptospires to adjust the orientation of their periplasmic flagella. From these findings, it is evident that the structure of leptospiral flagella is more sophisticated than previously thought, reinforcing the fact that other new proteins contributing to the motility of leptospires could be discovered in the near future.

## 5. Antigens Involved in Leptospiral Infection

A multitude of leptospiral antigens were successfully isolated and characterized for diagnostic and research purposes in the existing literature [120]. The classification of *Leptospira* largely depends upon the type of antigens expressed on their surface. In host systems, leptospiral antigens are primarily recognized by two receptors: toll-like receptors and NOD-like receptors [121]. Most of the anti-*Leptospira* antibodies are produced against the organism’s LPS, which are composed of polysaccharides, proteins and lipids [41]. Isogai et al. [122] documented that LPS in leptospires are antigenically active and are found to be unique amongst leptospiral serogroups. To reinforce, the O-antigen present in leptospiral LPS differs from one strain to another [123]. Therefore, isolating this particular antigen would greatly assist in the detection of specific leptospiral strains. In addition, Nally et al. [124] inferred that the synthesis of O-antigen may be specifically regulated by leptospires depending upon the animal host infected, further reinforcing their specificity.

Type-specific main (TM) antigen is another surface antigen found in leptospires. As its name implies, TM antigen is highly serovar-specific. A study by Adachi and Yanagawa [125] found that the presence of homologous TM antigen inhibits the microscopic agglutination of leptospires. In contrast, no inhibition was evident when heterologous TM antigens were utilized. From these findings, the authors inferred that TM antigen is present at the surface of *Leptospira* as it evidently participates in the microscopic agglutination of leptospires with antisera. Besides that, F4 is also another surface-specific fimbrial antigen that is involved in the process of haemagglutination [126]. This antigen was once assumed to be similar as TM antigen, but Adler et al. [127] found that both of these antigens produced unique results during immunodiffusion and haemagglutination tests. Moreover, F4 was found to cross-react widely amongst different serovar groups, whilst TM is strictly serovar-specific. The aforementioned findings further reinforced the contention that these two antigens are separate entities (with different antigenic identities) located at the surface of leptospires.

As documented by Guerreiro et al. [59], various leptospiral antigens are involved in the humoral immune response towards leptospirosis—namely, LipL32 and LipL4 in the outer membrane portion, heat shock proteins (GroEL and DnaK) in the cytoplasmic fraction and P37 in the periplasmic portion. GroEL and DnaK are heat shock proteins that are highly conserved in leptospiral species. These proteins provide unique responses in different strains and their high adaptability towards temperature shifts play a pivotal role in the virulence and infective potential of leptospires [128]. Natarajaseenivasan et al. [129] found that acute phase sera detect GroEL more frequently than other proteins, signifying its role during the acute phase of leptospirosis. As evident from increased levels of total IgG, IL-10 and lymphocytic proliferation, Atzingen et al. [130] demonstrated that the deliberate fusion of DnaK proteins with other leptospiral proteins promotes an enhanced immune response, as compared to the singular effects elicited by the individual proteins. Collectively, these findings accentuate the significance of each leptospiral antigen and their distinctive roles during the development of leptospirosis, allowing leptospires to easily infect and inflict harm against host systems.

## 6. Transmission and Pathogenesis

In the existing literature, several antigens have been reported as potential virulence factors of *Leptospira*, including the various outer membrane proteins, LPS, adhesion molecules and haemolysins. Although leptospires adopt a fairly linear framework of transmission, adhesion and cell entry into host cells, the exact molecular pathophysiology pertaining to leptospirosis is still a perplexing conundrum for many [131]; even so, significant amounts of notable research progress have been made recently. Martins-Pinheiro et al. [132] conducted an in silico search for DNA repair pathways in leptospires, allowing them to identify several important genes required for leptospiral infection. A multitude of key metabolic pathways in *Leptospira* have also been mapped out or further elucidated, including cobalamin biosynthesis and free radical detoxification [133]. Keeping up with the momentum from the aforementioned contributions, this section reviews the currently established sequential steps in the transmission and pathogenesis of leptospirosis as an attempt to disentangle, unearth and bring clarity to its rather complex mechanisms.

### 6.1. Transmission

Leptospires are primarily transmitted via two exposure routes; either by direct contact with an infected animal, or by indirect contact with environmental media such as soil and water that are contaminated with body fluids (especially urine) of infected animals [134]. Consumption of leptospiral-contaminated water, penetration through open wounds, abrasions and mucous membranes (conjunctival, oral, conjunctival or genital surfaces) are common portals of entry for the bacteria, ultimately bypassing the external host tissue barriers [14,22]. The corkscrew-like motility seen in leptospires allows them to move through more viscous barriers (including host connective tissues) fairly easily, contributing to their highly invasive nature [135]. Stern et al. [136] documented that accidental swallowing while swimming outdoors is a possible risk factor for leptospiral infection, as evident from the 2005 leptospirosis outbreak in Florida amongst adventurer race peers. In the existing literature, rodents [137], pigs [138], horses [139,140], cattle [141], dogs [142,143] and various wild animals such as opossums [19], deer [27] and pinnipeds [134] are some of the established carriers of *Leptospira*.

According to Levett et al. [144], leptospires have the tendency to inflict chronic renal diseases onto animal carriers; thereby explaining why the urine of *Leptospira*-infected animals contains large numbers of the organism. These bacteria tend to accumulate at the proximal convoluted tubules of their host, colonizing and multiplying rapidly from the get-go, whilst some others are released into the environment via urination [14]. Although it is well-established that leptospirosis is rarely transmitted from human to human through conventional means (hence the term zoonotic), de Oliveira et al. [145] have shown that it is possible for breast feeding to spread *Leptospira* from infected mothers to neonates. As reported by Harrison and Fitzgerald [146], sexual intercourse is also a possible transmission route (albeit rare) as there is still a small chance for *Leptospira*-contaminated urine to be exchanged directly between sexual partners. Infected rodents can also transmit the disease to livestock and pets, further expanding the disease transmission as livestock and pet borne. Overall, the tendency for each transmission route to occur depends upon the demographical, geographical, agricultural and livestock factors of a particular population [137].

### 6.2. Leptospiraemic Phase

The early (acute) stage of leptospiral infection is described using various terminologies, such as leptospiraemic [36], anicteric [24] and bacteraemic [147], all of which essentially denotes the same phase of infection. The sudden onset of myalgia, fever and headache are major manifestations of this phase [148]. There are also a few non-specific symptoms such as anorexia, nausea and abdominal pain, which are also seen in other unrelated diseases [147]. According to a study by Banfi et al. [149], leptospires were evidently found in the bloodstream just 10 min after deliberately infecting test animals via intra-peritoneal, intradermal and intra-ocular administrations. The twisting motion of periplasmic flagella plays a pivotal role in the aforementioned finding, allowing leptospires to enter the host bloodstream in mere minutes [24]. The spontaneous haematogenous dissemination of leptospires is in stark contrast to other spirochete species like *Treponema pallidum* and *Borrelia burgdorferi*, which prefers to establish localized infection in the skin and produce obvious lesions. Bacteraemia caused by leptospirosis is well-established to be very different from those caused by typical bacteraemic agents such as *Enterobacteriaceae*. Werts et al. [48] substantiated the aforesaid statement as they found that human TLR4 can easily recognize very low concentrations of LPS derived from *Escherichia coli*, but unable to do so in the case of leptospiral LPS. To reinforce, Que-Gewirth et al. [150] documented a very distinctive methylated phosphate residue found on the lipid A of leptospiral LPS, which is not found in other distinct species like *E. coli*. This allows leptospires to seemingly camouflage themselves from the host innate immune response, essentially providing them enough time to inflict damage before they get detected. Despite all of this, a study by Goris et al. [151] accentuated that the involvement of TLR2, TLR4 and TLR5 are still significant factors in host defense against leptospirosis, and further studies should be carried out to better elucidate the pathophysiological mechanisms of *Leptospira*.

### 6.3. Adhesion and Cell Entry

Although they are generally considered to be extracellular pathogens (mainly existing in the host bloodstream) [28], leptospires are equipped with an armament of virulence factors, allowing them to easily adhere, enter and replicate within host cells. As such, various studies in the existing literature have suggested that pathogenic leptospires should at least be considered as transiently intracellular, since they were evidently found to replicate and survive within macrophages and other non-phagocytic cells [28,152,153]. Spontaneous and recurrent damage in the endothelial lining of small blood vessels is considered to be the major pathological phenomena of leptospirosis, but the exact mechanism as to how leptospires penetrate the endothelium is not fully understood. Barocchi et al. [154] managed to infer that leptospires have the ability to translocate between cells through polarized monolayers, but unable to do so through cell junctions. Through in vitro studies, Banfi et al. [149] found that various proteins are involved in leptospiral adhesion to cells; leptospiral proteins can bind not only to ECM components (fibronectin, elastin, laminin and collagen), but they can also adhere to complement regulators and plasminogen by using LigB and LipL32. As reviewed by Murray [155] and Fernandes et al. [156], a total of 34 and 17 leptospiral proteins were reported to be capable of binding to fibronectin and plasminogen, respectively. The multifunctional aspect of the aforementioned proteins explicitly highlights the sophisticated nature of the mechanistic and pathophysiological framework in leptospires.

According to Liu et al. [157], adhesion-related receptors of leptospires were hypothesized to be localized at their cell terminals (similarly like *Treponema denticola*). When incubated with J774A.1 cells, leptospires tend to bind to these cells at both ends, forming a dumbbell-like shape. In contrast, incubation with Vero cells did not produce the dumbbell shape as previously described, as the Vero cells only bind to one end of the leptospires. This coincides with their inference that the pattern of attachment seems to be distinctively varied according to the cell lines utilized. This particularly unique fashion of adhesion provides leptospires with a somewhat “customizable” form of cell internalization, ultimately becoming an important factor of their virulence. A homologue of mammalian cell entry (Mce) protein, which is normally found in *Mycobacterium tuberculosis*, are also found in pathogenic leptospires [158]. These proteins are documented to confer leptospires with the ability to adhere to host cells, as inferred from studies done on recombinant Mce-bound integrins [159]. With so many adhesin proteins at their disposal, leptospires are capable of binding to various cells, including endothelial cells, monocytes, kidney epithelial cells and fibroblast cells [41]. As such, these bacteria can colonize, replicate and survive in wide variety of tissues throughout the host system. Liu et al. [157] also inferred that the internalization of leptospires is via receptor-mediated endocytosis. They found that infected cell lines contain numerous leptospiral-laden phagolysosomes, an observation that later contributed to the suggestion that these pathogens could be residing in phagolysosomes for their continual replication and survival. This is reinforced by the fact that leptospires reside temporarily within cells as an effort to avoid complement and antibody-induced killing [41].

### 6.4. Weil’s Disease

**Figure 4:**
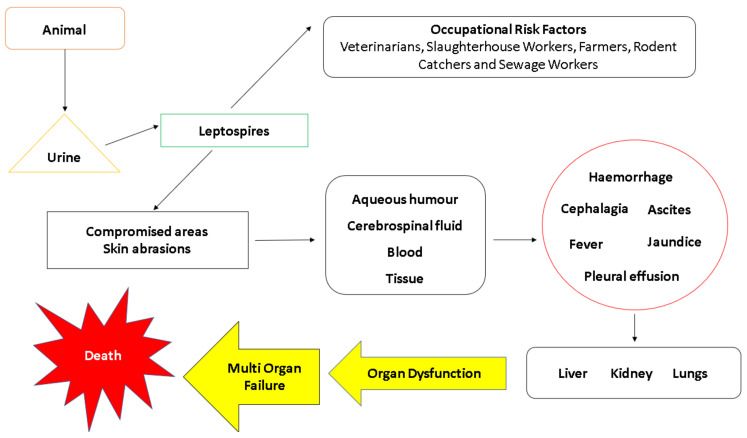
Summary of the transmission and pathogenesis framework in leptospirosis.

If leptospirosis is not promptly or adequately treated during the acute phase, leptospires in the bloodstream can sometimes translocate even further to distant host tissues and the disease slowly worsens. This particular phase is commonly known as Weil’s disease [24], which is an extremely severe form of leptospirosis. It is also known as the late phase [147], leptospiruria [36] and icteric phase. Essentially, the aforesaid terminologies denote the progression of leptospirosis into a very severe systemic disease, highlighting the tendency for leptospires to inflict system-wide damage that can be detrimental to health. Documented to last for up to 30 days, the immune phase typically begins from 7–10 days after the first onset of symptoms [36]. Satiya et al. [160] emphasized that untreated leptospirosis can lead to fatal hepatic manifestations, such as jaundice. Liver failure, kidney failure and respiratory shock are also notable complications of severe leptospirosis. As the disease remains untreated for a long period of time, the organisms become highly invasive as large numbers of cell membrane-degrading enzymes are actively secreted. Other proteins produced during this phase includes orthologs of hemolysin, proteolytic enzymes like collagenase, metalloprotease and thermolysins, which are all necessary for degradation of ECM components and assists their invasion [161]. As such, the host immune system responds by producing large amounts of cytokines and recruiting numerous white blood cells, but these efforts are futile against leptospires, easily leading to multi-organ failure and even death. A summary of the transmission and pathogenesis framework of leptospirosis is illustrated in (Figure 4).

### 6.5. Evasion of Host Immune System and Virulence Factors

Despite ongoing research efforts, the pathophysiological mechanism of leptospirosis remains poorly understood. However, it is theorized that this disease occurs as a result of the host immune response towards the organisms, which by now should be excessively abundant in the blood, liver, lungs, kidneys, cerebrospinal fluid and the aqueous humour among other organs [160]. As leptospires bind to host cells, cytokines (interleukin-6, interleukin-10 and TNF-α) and antimicrobial peptides (AMPs) are released to limit the invasive damages incurred by the bacteria. In response, phagocytic cells engulf the organisms; however, as described previously, leptospires are capable of replicating and surviving in phagolysosomes. The host immune system continuously releases excessive amounts of cytokines, culminating as a destructive response instead of a beneficial one [162].

Their high motility and resistance to complement proteins are noted to be effective evasive mechanisms in leptospires [149]. Other virulence factors documented in the existing literature includes their ability to form biofilms, high stability of their outer membrane [24], active LPS biosynthesis, iron uptake, innate stress response and collagenase activity [28,159]. Two-Component System (TCS) proteins are also reported to be responsible for leptospiral virulence. Adhikarla et al. [163] reinforced the aforementioned statement as they found that leptospiral strains in hamster models become avirulent after deliberately disrupting two TCS genes—namely, *lvrA* and *lvrB*. The authors concluded that much of the organism’s virulence stems from sophisticated signalling pathways that are yet to be unravelled. As it remains uncertain, genetic, proteomic and transcriptomic research is definitely required to further understand the progression and pathogenesis of leptospirosis.

## 7. Co-Infections

The tendency for leptospirosis to co-exist with other diseases presents as a diagnostic and therapeutic challenge for many. This creates significant amounts of difficulty in pinpointing the singular diseases, and provision of too many medications could be detrimental to a particular patient. This brief section provides an overview regarding the aforesaid challenges to acknowledge the existence of leptospiral co-infections and to establish a better understanding of their tendency to co-infect.

Dengue is often associated together with leptospirosis. Several leptospirosis/dengue co-infections have been reported in various countries, including Puerto Rico [164]. A study by Sachu et al. [165] found an association between leptospirosis/dengue co-infection with symptoms such as bleeding gums and rashes. Rainfall was also found to have a positive correlation with leptospirosis/dengue co-infection, but their results were deemed statistically insignificant. Diagnosing and treating this co-infection in the early phases can be quite challenging. as both dengue and leptospirosis evoke clinical manifestations that are similar to acute febrile illnesses. However, the actual problem lies in their combined severity; to reinforce, severe leptospirosis can lead to jaundice and multiple organ dysfunction, whilst dengue can lead to dengue haemorrhagic fever (DHF) and dengue shock syndrome (DSS) [166]. Treatment of leptospirosis is antibiotic-focused, while dengue is often treated symptomatically. However, the overlapping spectrum of clinical manifestations can easily lead to misdiagnosis, subsequently delaying provision of appropriate antibiotics and symptomatic care [165].

Zika virus (ZIKV) disease is a mosquito-borne malady that often leads to congenital abnormalities, including microcephaly [167]. Reports of leptospirosis/Zika co-infections are evident in the existing literature. Neaterour et al. [168] reported a case of leptospirosis/Zika co-infection without any obvious clinical picture. The patient did not have any distinct rashes (obvious picture of ZIKV infection) but ZIKV nucleic acid levels were notably increased. The authors theorized that the symptoms of ZIKV infection may be “masked” by the more virulent symptoms inflicted by leptospirosis (presenting with more obvious signs and symptoms). Biron et al. [169] found that the tendency for this co-infection to occur highly depends on geographical factors; prevalence is notably higher in returning travellers/tourists from tropical regions.

Leptospirosis/malaria is an emerging co-infection especially prevalent in the borders of Thailand and Myanmar. Fever is usually the first symptom detected in afflicted patients, with no obvious signs of leptospiral involvement. However, as the co-infection develops further, clinical pictures of leptospirosis becomes more apparent as Weil’s disease, pulmonary haemorrhage and/or uveitis develops. Diagnosing the co-infection at this stage could prove to be futile due to the combined severity of both diseases. An alarming fatality rate of approximately 1–14% coincides with poor diagnosis associated with the co-infection; to reinforce, diagnosis attempts often result in negative microscopic agglutination test (MAT) results, even though the patient’s serum contains high levels of anti-leptospiral IgM. To make matters worse, the emergence of multi-drug-resistant (MDR) malaria hinders therapeutic efforts, resulting in poor prognosis [170].

According to a case report by Markotić et al. [171], a Croatian soldier was suspected of suffering from leptospirosis and Hantavirus haemorrhagic fever with renal syndrome (HFRS). From his background, the soldier lives in a rodent-infested area, thereby increasing his likelihood of contracting the co-infection. Clinical pictures that were reported include fever, headache, visual difficulties, renal failure, conjunctival suffusion and petechiae. Diagnosing the patient via conventional methods is difficult as a multitude of symptoms (renal failure, pulmonary disorders and liver disorders) are quite similar. In light of that, leptospirosis was positively diagnosed via microagglutination whilst Dobrava-Belgrade orthohantavirus (DOBV) was identified as the causative agent (for HFRS) via PCR analysis. Although HFRS is classified as a noncurative disease, the severity of leptospirosis can be mitigated with proper and monitored use of antibiotics. Sunil-Chandra et al. [172] reported a similar co-infection with Puumala virus (PUUV) as the causative agent. Gamage et al. [173] also documented a fairly similar co-infection caused by Thailand virus (THAIV). Overall, the prompt identification of their role in immune responses and the ability to differentiate the two diseases are of utmost importance for the betterment of treatment.

Another common co-infection is leptospirosis with scrub typhus, especially prevalent amongst agriculture workers. The non-specific nature of the collective symptoms reported (fever, headache, myalgia and conjunctival suffusion) persists as a problem for diagnosis and therapy. As scrub typhus can be seen mostly in Asia, South Pacific and Northern Australia, it can be inferred that the co-infection is also found majorly in these areas [174,175]. According to a report by Watt et al. [175], difficulties in diagnosing the co-infection led to the death of an agricultural worker (due to adult respiratory distress syndrome), further highlighting the severity of leptospirosis co-infections and its worrisome trend worldwide.

## 8. Clinical Manifestations

**Figure 5:**
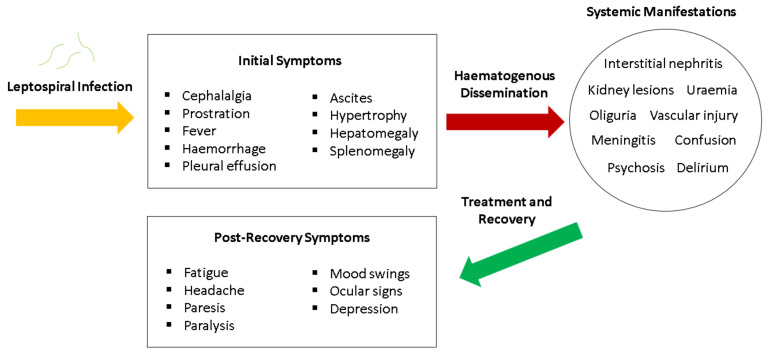
Summary of the clinical manifestations seen in leptospirosis.

In humans, it is well acknowledged that the symptoms seen in leptospirosis can be somewhat confusing and subtle, easily leading to diagnoses that are false negative. Lau et al. [147] documented that the mass majority of misdiagnosis in leptospirosis is largely due to the non-specific nature of the clinical manifestations seen in the disease. The initial symptoms that are documented in the existing literature includes cephalalgia, prostration, fever, haemorrhage, pleural effusion, ascites, hypertrophy, hepatomegaly and splenomegaly [22,24,148,176,177]. Although these manifestations are deemed to be associated with leptospirosis, they should not be exclusively denoted as confirmatory signs of the disease. The non-specific and subtle nature of these symptoms highlight the severity of leptospirosis, as many of these signs can go unnoticed by patients and physicians alike. Furthermore, this disease inflicts harm to multiple organs, possibly leading to interstitial nephritis, kidney lesions, uraemia, oliguria, vascular injury, jaundice, meningitis, confusion, psychosis and delirium [22,24,148,176]. After patients are sufficiently treated with care, they tend to experience post-recovery symptoms, most notably fatigue, headache, paresis, paralysis, ocular signs, mood swings and depression. These signs denote the persistence of leptospires in the patients, which are seemingly “camouflaged” by the host immune response. A simple graphical presentation summarizing the aforementioned manifestations is outlined in Figure 5. In pregnant women, leptospirosis can sometimes cause congenital leptospirosis, stillbirth, foetal cardiotocography changes and even foetal death [178]. Overall, the lack of classical symptoms and its tendency to exist as co-infections with other unrelated diseases highlights the importance of highly specific diagnostic measures for leptospirosis, which are discussed in the forthcoming section.

## 9. Diagnosis of Leptospirosis

In the early stages, physicians are often unaware that their patients are infected with *Leptospira*. Diagnosis is only often made when clinical symptoms depicting Weil’s disease, pulmonary haemorrhage, jaundice or renal failure are apparent. The differential diagnosis for these symptoms is confounding and ranges from benign viral syndromes of childhood to meningitis and sepsis [179,180]. Sometimes the severity also varies depending upon the individual. Few individuals may possess the antigens of the particular serovar of the *Leptospira* species but may not show or be void of any symptoms of the infection. Hence, the diagnosis of leptospirosis must not be done by the clinical symptoms shown by the patients but rather by laboratory diagnosis. More effective and accurate diagnostic tools have been developed in order to confirm the presence of a leptospirosis infection. The currently established diagnostic tools for leptospirosis include serological tests (microscopic agglutination test (MAT), solid phase assay, enzyme linked immuno-sorbent assay (ELISA) and indirect haemagglutination assay), direct diagnostic methods (microscopy, particularly phase contrast or dark field microscopy, histochemical staining and immunostaining), culture methods and molecular techniques such as nested polymerase chain reaction (PCR) [181]. Along with these techniques, scientists and researchers have also been developing other advanced techniques such as flow cytometry. Future advancements and refinements of the current diagnostic tools will definitely hasten the diagnosis process of leptospirosis, even in the early stages [182]. The aforementioned diagnostic techniques are discussed in detail here under.

### 9.1. Clinical Findings

The clinical findings of leptospirosis are mostly based on the increment or decrement in the levels of enzymes, blood cells or any bodily contents. These findings allow physicians to evaluate homeostatic changes in the patients’ body when it is going through leptospirosis infection [177,180]. Documented samples include blood, cerebrospinal fluid and urine [183]. Erythrocyte sedimentation rate (ESR) is typically elevated due to inflammation and white blood cell count (WBC) ranges from below average to above average. Liver function test (LFT) usually shows an elevation in amino transferase, bilirubin and alkaline phosphatase. In cases of severe leptospirosis, hyperbilirubinaemia is evident due to jaundice (causative). Manifestations may be variable from mild to severe hepatic dysfunction and hepatomegaly. The most affected liver functions are bilirubin metabolism and protein synthesis [184]. Renal function test (RFT) typically shows elevated plasma creatinine in patients with severe leptospirosis. Histological findings of *Leptospira*-infested kidneys include tubule-interstitial nephritis, interstitial fibrosis and tubular atrophy. Proximal tubule dysfunction and hypokalemia can also be observed in adult male workers affected with leptospirosis [79]. Urine analysis demonstrates proteinuria, pyuria, microscopic haematuria, hyaline and granular casts [185]. Lumbar puncture shows an elevated cerebrospinal fluid pressure, predominance of lymphocytes and polymorphs [186]. Peripheral blood smear typically shows peripheral leukocytosis with obvious left shift and thrombocytopenia [187].

### 9.2. Serological and Indirect Diagnostic Methods

For many years, serological diagnosis or serology has been considered to be the cornerstone in identifying leptospiral infections. Serological studies are done on serum samples extracted from individuals suspected of suffering from leptospirosis. Usually, these studies are based on the detection of specific antibodies against various leptospiral antigens. Since leptospires have a very long doubling times in culture and the culture takes weeks to grow, the diagnosis of leptospirosis mostly depends on serological results.

#### 9.2.1. Microscopic Agglutination Test (MAT)

MAT has been widely used for the diagnosis of leptospirosis through detection of antibodies produced against the antigens of *Leptospira* serovars [188]. This technique utilizes live bacterial cultures and is routinely performed by incubating patient’s serum with various serovars of *Leptospira* [189]. MAT titre is obtained by testing various serum dilutions with a positive serovar. A four-fold rise of MAT antibody titre is a definite evidence of *Leptospira* infection. Regarded as the *gold standard* for all diagnostic techniques, this assay has a high sensitivity and allows for the detection of group-specific antibodies [190,191]. In regions where leptospirosis is common, there may be a substantial proportion of the population with elevated titres of MAT. In addition to this, the serum from patients may react with a different serovar than the infected one. In case of many numbers of samples, performing MAT would be very difficult as it is a complicated test. Moreover, diagnostic laboratories are also required to have all the circulating types of *Leptospira* serovars, which may be costly [192]. It would not be useful during the early stages of the disease as the antibodies against the leptospires are usually not present, or if at all present, it will be at an extremely low level in the cerebrospinal fluid [180]. Hence, other diagnostic techniques have been developed that are more rapid and easier to carry out.

#### 9.2.2. Microsphere Immunoassay (MIA)

Although MAT has been the *gold standard* and most widely used technique in the diagnosis of leptospirosis, it relies heavily on live cultures and subjective interpretation of results. This technique is also unable to clearly differentiate between the classes of anti-leptospiral antibodies. MIA (which utilizes the Luminex xMap technology) is able to positively diagnose samples from those that were previously deemed non-reactive. It is also capable of differentiating between IgM and IgG antibodies against *Leptospira.* The MIA test is carried out by preparing antigens from pure *Leptospira* cultures and preparing immunoassays for IgG and IgM [191]. Briefly, the technique relies on magnetic-coated polystyrene beads filled with bi-coloured fluorescent dyes in different ratios resulting in 500 distinct bead sets. Each bead set may be coated with a different antigen to allow simultaneous measurement of antibody response to up to 500 different antigens. This high throughput screening system allows processing of high numbers of patient samples per day [193]. Its speed, sensitivity and accuracy of multiple binding events measured in the same small volume have the potential to replace many diagnostics methods and deliver hundreds of analyte data simultaneously. The test has shown notable success in identifying antibody types as well as the reactivity of antigens. Additional benefits of using MIA to diagnose leptospirosis include significant cost-reduction and allows for bulk sampling against serovars at one time.

#### 9.2.3. Enzyme-Linked Immunosorbent Assay (ELISA)

**Figure 6:**
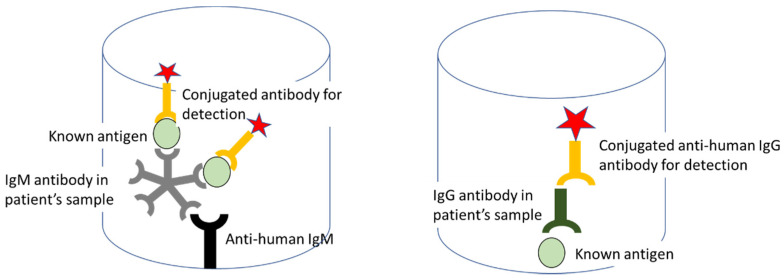
ELISA using IgM and IgG antibody for leptospiral diagnosis.

ELISA can also be used to diagnose leptospirosis by utilizing leptospiral-specific IgM and IgG [194] from sera of patients infected with different leptospiral serovars. According to a study by Hartman et al. [195], subjects with leptospirosis produced specific IgM and IgG antibodies that are detectable by ELISA, even with low titre of antigens in their serum. Only a few subjects had IgG agglutinins whereas all of them produced IgM agglutinins. The specificity and sensitivity of the test suggests that the ELISA anti-IgM technique is a suitable method for detecting leptospiral antibodies in sera for diagnostic and epidemiological purposes [196]. The specificity of the antisera used to prepare the conjugates was confirmed by immunodiffusion and by immunoelectrophoresis against purified human IgM and IgG immunoglobulins [197] (Figure 6). While it is indeed effective, Shekatkar et al. [198] documented that antibody levels are generally low or absent during early phases of the infection. This can be a problem in diagnostic terms, as this could easily lead to false negative diagnoses [199,200].

#### 9.2.4. Indirect Haemagglutination Assay (IHA)

**Figure 7:**
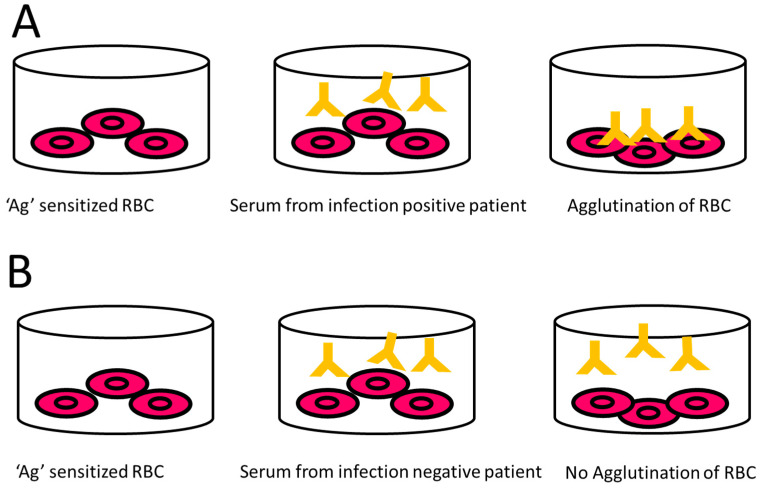
Illustration depicting the principles of IHA to detect leptospires (**A**) positive reaction and (**B**) negative reaction.

IHA also detects IgM and IgG produced against *Leptospira* antigens in the body after bacterial entry (within 4–6 days) [194]. Commercial kits for this assay are widely available in the market, following the principle as illustrated in (Figure 7). Levett and Whittington [201] documented that IHA has a diagnostic sensitivity of 92% and a specificity of 95%. This assay can be utilized as an initial diagnostic tool for patients who are clinically suspected to be having acute leptospirosis. It is advantageous due to its relatively low cost and it requires no specialized equipment or any strict incubation conditions. One significant disadvantage of using IHA is that the results may not be interpretable when there is non-specific haemagglutination [201]. Bajani et al. [202] documented that IHA is significantly less sensitive than ELISA for the diagnosis of leptospirosis. It was also found that the sensitivity of IHA is somewhat similar to that of MAT assay [181].

#### 9.2.5. Dipstick Assay

**Figure 8:**
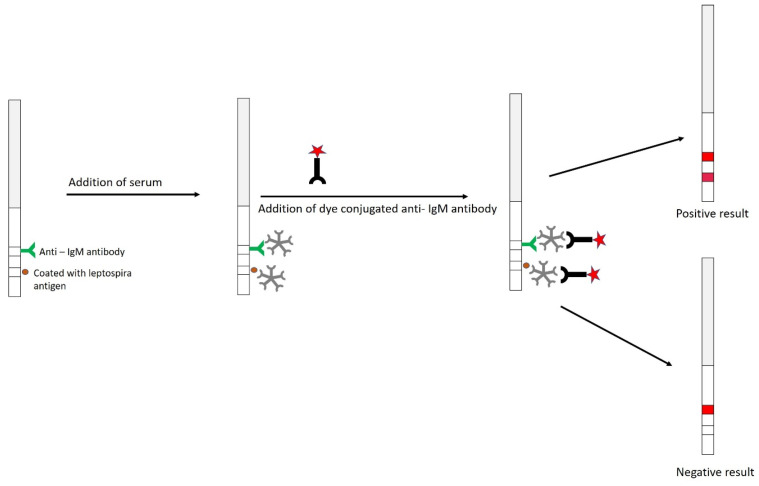
Illustration depicting dipstick assay.

Dipstick assay is an easy and robust technique that allows for rapid screening and diagnosis of patients suspected of having leptospirosis. The LEPTO dipstick test is a rapid field test for leptospirosis that does not require special laboratory equipment or well-trained personnel [203,204]. This assay (tested under different epidemiological and clinical conditions) demonstrated high sensitivity, specificity and predictive values. The dipstick assay, as described by Hatta et al. [203], assesses the samples using a dipstick which contains two horizontal bands—namely, the lower band consisting of broadly reactive specific antigens and the upper band which acts as an internal control as it consists of antihuman IgM antibodies (Figure 8). Bound IgM antibodies are detected in non-enzymatic reactions with a stabilized anti-human IgM dye conjugate and the sensitivity is comparable to IgM-ELISA [181].

#### 9.2.6. Flow Cytometry (FCM)

The purpose of using FCM for diagnosing leptospirosis is due to its high sensitivity towards the size and shape of leptospires [205]. The scattering parameters—namely, forward scatter (FSC) and side scatter (SSC)—play a pivotal role for this technique. FSC is associated with the cell size and optical refraction index of the outer membrane, whilst SSC is related to the bacterial granularity [206]. Diagnosis of leptospirosis can be done by assessing the light scattering patterns after carrying out the agglutination reaction between the antigen and antibody of a specific serovar type in *Leptospira*. Analysis is possible because recent flow cytometers have a resolution capacity to detect and observe particles that are less than 0.5 µm diameter [207]. These analysers are usually equipped with an excitation light source that emits laser beam from it at a particular wavelength, an amplifier to amplify the signal and detectors such as a photodiode (for FSC) or a photomultiplier (for SSC) to detect the amplified signal [208]. According to Yitzhaki et al. [209], FCM is more efficient, rapid (can be completed within 1.5 h), specific and sensitive for the diagnosis of leptospirosis, especially in identifying specific serovars during the acute phase of the disease.

### 9.3. Direct Diagnostic Methods

#### 9.3.1. Microscopy Techniques

This technique is particularly useful for observing leptospires in culture, particularly when they are present in large numbers, and also for observing the agglutination formed via MAT (microscopic agglutination test). Leptospires present in patient samples can be concentrated using centrifugation. One of the disadvantages in using microscopy for diagnosis is that the direct microscopic observation of leptospires in urine (leptouria test) may have a low specificity since the presence of fibrin and protein in the urine samples can be mistaken for leptospires [180]. As such, false diagnosis is possible. Phase contrast microscopy is useful in observing transparent, colourless and/or unstained specimens which can be referred to as “phase objects”. Even though phase contrast microscopy is useful for visualizing leptospires in the laboratory, its viability in the diagnostic laboratory is clouded by its technical limitations in thick suspensions and its optical characteristics [210] Leptospires can be easily detected under dark field microscopy as thin, coiled, motile organisms in blood and urine samples of patients with leptospirosis. However, the positivity of dark field microscopy decreases from 100% to 90.9% as the duration of infection increases for more than one week [211]. Another disadvantage of this technique is that both false positive and false negative diagnosis can be easily made, even by experienced technicians.

#### 9.3.2. Staining Techniques

A variety of histopathological stains are used for the detection of leptospires in clinical specimens. Although silver stains have been documented to be of use in the literature [212], the Warthin-Starry stain is widely in use now. Azizi et al. [213] commented that histopathological stains (including Warthin-Starry stain) can sometimes confer false negative results, as the leptospirosis burden in tissue biopsies (such as kidneys) may not be significant. Besides histopathological stains, immunohistochemical assay and immunoglobulin fluorescent staining are also documented to be useful diagnostic tools for leptospirosis. Immunoglobulin staining is usually done on tissues with positive immunoreactivity towards leptospiral antigens [214]. The technique revolves around the use of enzymatic or metallic labels on secondary antibodies. Phosphatase, peroxidase or metallic gold-labelled antibody can be used in a variety of formats to stain leptospires in clinical specimens [215]. This technique has the advantage of being useful with formalin-fixed tissue. It can also be used to detect leptospires even when their numbers are significantly low, or when there are materials that precludes the use of dark field microscopy. However, immunostaining requires a primary antibody that is specific for the serovar being sought [180]. Too many serovar varieties in a pool would dilute any one, so high-titre antisera conjugates are required. In other words, it may be not be advantageous in early infections. This is not widely used as a primary diagnostic tool in recent studies.

#### 9.3.3. Culture Technique

In this method of diagnosis, samples from a suspected patient, usually urine and/or blood sample, are taken and streaked onto a culture flask containing fluid media (generally used for primary culture). Oleic acid-albumin media of EMJH is the most commonly used media for this purpose. It is composed of a basic medium which consists of ammonium chloride, thiamine, disodium phosphate and monopotassium phosphate as well as various enrichment factors including Tween 80 and albumin [216]. Antibiotics such as rifampicin, neomycin, actidione can be added to the media for selective isolation of bacteria from contaminated samples [217]. Advantageously, leptospires can be cultured from blood or cerebrospinal fluid samples during the acute phases of the infection (lasts for about 10 days). Even though this method provides highly accurate results, it is a tedious and long process. This is because leptospires take a very long time to divide (doubling time is estimated to be 6–8 h) and the whole culture can take almost 3 months to grow. As leptospires are highly infectious organisms, they need to be handled with utmost care, even by experienced personnel. Therefore, there may be risk of laboratory-acquired infections with this technique [180].

#### 9.3.4. Polymerase Chain Reaction (PCR)

PCR is used to amplify DNA content as an essential pre-requisite for sample analysis. It is especially important when the DNA content in samples is deemed to be low or undetectable. With PCR, *Leptospira* can easily be detected from urine samples or blood samples during the early stages of the disease [218]. During acute leptospirosis, the antibody titre may not be high enough for accurate serological diagnosis. A prime advantage of using PCR is that results are generated very quickly, as compared to conventional techniques like culture [219,220,221]. With specially designed primers, a variety of leptospiral targets can be amplified for diagnosing the disease—namely, 16S ribosomal RNA, various pathogenic wild-type genes and mutated genes. Standard PCR procedures require the subsequent use of agarose gel electrophoresis to detect target leptospiral genes [222]. However, real-time PCR (RT-PCR) is capable of providing diagnoses results immediately after the DNA content is amplified. According to Merien et al. [223], RT-PCR is highly sensitive and specific, providing accurate results in the long run. One of the major drawbacks of RT-PCR for diagnosing leptospirosis is that the primers may sometimes bind to a non-specific site, leading to false positive results. Hence, it is recommended that more than one technique should be used to increase the diagnostic sensitivity and specificity. This can be performed by developing real-time multiplex PCR assays. Multiplex PCR is done by using two sets of primers instead of one in order to increase the specificity of DNA binding and amplification [88]. The use of more than one target may be important to distinguish between true and false-positive PCR results. Nested PCR also helps in detecting more specific and sensitive DNA sites using additional sets of primers [224,225,226].

PCR may also prove to be useful when diagnostic resources are limited or scarce. Conventional diagnostic techniques such as culture can be useful, but most of its resources (if not all) are not available in most countries [227]. As current diagnostic trends for leptospirosis strongly suggest the use of both serological and molecular techniques for heightened accuracy, the combinatorial use of PCR and ELISA (in place of the cumbersome *gold standard* MAT) for early diagnosis is a powerful alternative in resource-poor countries [228], highlighting the significance of combinatorial techniques in diagnoses. Besides that, Koizumi et al. [229] developed a revolutionary loop-mediated isothermal amplification (LAMP) technique to detect a 16S rRNA gene (*rrs*) that are typically found in urines of patients infected with pathogenic leptospires. This technique also caters well for developing countries as it is cost-effective, rapid and highly accurate in producing consistent results. To summarize, the value of PCR in clinical diagnosis is unparalleled. Further upgrades and advancements of current PCR techniques (as well as other revolutionary techniques such as LAMP) could expand these techniques as routine tests for leptospirosis.

## 10. Epidemiological and Transmission Patterns of Leptospirosis: Future Concerns

Although the risk factors and transmission of leptospirosis have already been elaborated in previous sections, the erratic epidemiological and transmission pattern of the disease is slowly culminating into a global concern, and is thus separately highlighted here. It is evident that various epidemiological characteristics coincides with higher prevalence of leptospirosis cases. Blasdell et al. [230] suggested that urbanization potentiates the transmission and circulation of *Leptospira* spp., resulting in substantial public health crisis as the “possibility” for leptospirosis to occur in urban areas are typically underestimated. Due to various factors such as overcrowding and contact with animals [8,9], leptospirosis is especially prevalent in rural areas, such as Senegal in West Africa [231]. Nozmi et al. [232] also found that a rural community in Malaysia has significantly low knowledge, attitude and practice (KAP) elements towards leptospirosis, coinciding with high prevalence of leptospirosis cases. Places with high rates of natural disasters (such as major floods) [10], multiple records of international travels [22] and highly engaged with recreational activities such as water-based sports [24] also coincides with high cases of leptospirosis.

The transmission patterns of leptospirosis outbreaks vary by a huge margin. As reviewed by Haake and Levett [134], cases of leptospirosis endemics are especially prevalent in tropical areas (peak incidence at high temperatures), often culminating to large, isolated epidemics and outbreaks following rainfalls and flash floods. The disease is highly seasonal-dependent, as temperature is a limiting factor for the survival of leptospires. This highlights the tendency for leptospirosis cases to increase dramatically with certain seasons, climate changes and natural disasters [10]. Besides that, a systematic review by Bierque et al. [233] highlighted the significance of biofilm formation in the transmission of leptospires. To reinforce, pathogenic leptospires are capable of surviving in biofilms in natura (even in nutrient-free settings), and their tolerance towards antibiotics can be increased by 5-fold. From this, it is evident that biofilm formation significantly favours leptospiral survival and persistence, ultimately potentiating their transmission rates. As such, sustained outbreaks are common [10,12], even extending to broad geographical regions. As the epidemiological and transmission patterns of leptospirosis remain unprecedented to many, various global concerns may arise in the near future, possibly leading to pandemics and more deaths. This further highlights the importance of epidemiological data in an effort to combat infectious diseases worldwide, a feat that is not possible without the collaborative efforts of many.

## 11. Conclusions

From this review, it is clear that various risk factors increase the likelihood for one person to be inflicted with leptospirosis, but these factors can be unpredictable at times. Pathogenic leptospires attain their virulence due to the presence of various proteins (especially outer membrane proteins), allowing them to transmit and cause diseases easily. The unrelenting nature of *Leptospira* leads to a deadly combination of clinical symptoms, and often times these treatments may be insufficient. The recent emergence of leptospiral co-infections with unrelated diseases such as HFRS, dengue and malaria highlight the culminating severity of leptospirosis. Diagnosis of leptospirosis has always been difficult.

## Figures and Tables

**Table 1:** Number of cell layers based on the leptospiral strains.

Strain	No. of Layers	Reference
*L. pomona*	3 layers (dense-light-dense)	[113]
*L. icterohaemorrhagiae*	5 layers (dense-light-dense-light-dense)	[115]
A large spirochaete found in ANU-G lesions	5 layers (dense-light-dense-light-dense)	[116]

## Data Availability

No new data were created or analyzed in this study. Data sharing is not applicable to this article.
